# Diagnostic Challenges in Fahr’s Disease: A Rare Case of Extensive Basal Ganglia Calcifications

**DOI:** 10.7759/cureus.91056

**Published:** 2025-08-26

**Authors:** Peter N Rodenko, Tamara Slavkovska, Vikrant Bhatnagar, Jada Williams, Deniz Dolun, Marienne Hamra, Edward Chai

**Affiliations:** 1 Internal Medicine, St. George's University School of Medicine, St. George, GRD; 2 Neurology, St. George's University School of Medicine, St. George, GRD; 3 Internal Medicine, The Brooklyn Hospital Center, Brooklyn, USA; 4 Neurology, The Brooklyn Hospital Center, Brooklyn, USA

**Keywords:** early onset fahr's disease, fahr's, fahr's disease, fahrs disease hypoparathyroidism, fahr’s disease or fahr’s syndrome, fahr´s syndrome, fahr's syndrome hypocalcemia, primary familial brain calcification (pfbc)

## Abstract

Fahr’s disease, or primary familial brain calcification (PFBC), is a rare neurodegenerative disorder characterized by bilateral intracranial calcifications, often involving the basal ganglia, cerebellum, and subcortical white matter. Clinical manifestations may include seizures, cognitive decline, movement abnormalities, and psychiatric symptoms. Although Fahr's disease is often idiopathic with an autosomal dominant inheritance, similar imaging findings may arise from non-hereditary secondary causes such as metabolic, infectious, or toxic conditions. Identifying the underlying etiology is essential for guiding management. We present the case of a 48-year-old African-American male with a history of intellectual disability, psychiatric illness, and gait instability, who was brought to the emergency department after a witnessed generalized tonic-clonic seizure. Non-contrast computed tomography (CT) imaging of the head revealed extensive bilateral calcifications involving the basal ganglia, cerebellum, and cerebral white matter. Laboratory findings showed marked hypocalcemia, low parathyroid hormone levels, and borderline elevated phosphate. No history of prior neck surgery or known familial endocrine disorder was reported. The patient was treated with calcium and vitamin D supplementation, antiepileptic therapy, and continued psychiatric management. He was discharged to subacute rehabilitation (SAR) with plans for follow-up in neurology and endocrinology clinics. This case illustrates the diagnostic complexity of intracranial calcification syndromes. While certain clinical and imaging features raised suspicion for idiopathic Fahr’s disease, the presence of metabolic abnormalities suggested a secondary etiology related to long-standing hypoparathyroidism. The absence of a definitive family history and social support, together with the chronicity of symptoms, added further ambiguity to the diagnostic picture. Whether the calcifications resulted primarily from an idiopathic process or developed in the context of longstanding, unrecognized hypoparathyroidism remains uncertain, though the latter was the leading hypothesis. A complete workup, including metabolic investigation and genetic evaluation when feasible, is critical in such cases. In patients presenting with seizures and neuropsychiatric symptoms alongside extensive intracranial calcifications, both idiopathic and secondary causes of Fahr’s disease should be considered. This case highlights the importance of integrating clinical, biochemical, and imaging data to guide diagnosis and management. Early identification and treatment of modifiable factors, even when the etiology is unclear, may improve patient outcomes.

## Introduction

Fahr’s disease, also known as primary familial brain calcification (PFBC), is a rare neurodegenerative disorder characterized by idiopathic dystrophic calcium deposits in various brain regions such as the basal ganglia, cerebellar dentate nuclei, and subcortical white matter [1,2]. The disease typically manifests in mid-adulthood but has a variable age of onset [3,4]. The true prevalence of Fahr’s disease remains uncertain due to its varied clinical presentation and frequent underdiagnosis. However, it is estimated to affect fewer than one in 1,000,000 individuals worldwide and is often identified incidentally through neuroimaging [2,5]. Fahr’s disease occurs idiopathically by definition, but may also be seen secondary to an identifiable metabolic, infectious, or genetic cause, which some in the literature refer to as Fahr’s syndrome [6]. 

Fahr’s disease is typically inherited in an autosomal dominant pattern and has been linked to mutations in the* SLC20A2* gene, which encodes a sodium-phosphate transporter involved in brain phosphate homeostasis [4,7]. Clinically, patients may present with a spectrum of symptoms including movement disorders, gait instability, seizures, cognitive decline, and mood disturbances, which correlate with the most commonly calcified brain regions in this disorder [6]. Psychiatric symptoms such as depression, psychosis, or executive dysfunction precede neurologic findings, leading to misdiagnoses of a primary psychiatric disorder on symptomatic onset [6]. 

Non-contrast computed tomography (CT) remains the most sensitive tool for detecting the hallmark bilateral calcifications in affected brain regions [2,5]. No definitive or disease-modifying treatment currently exists outside of symptomatic management. Laboratory workup is essential to distinguish primary from secondary forms. In this report, we present a case of a patient who was admitted with a witnessed seizure and was subsequently found to have radiographic findings consistent with Fahr’s disease.

## Case presentation

This case involves a 48-year-old African-American male with a past medical history of unspecified intellectual disability, psychiatric disorder (on quetiapine), and gait instability, who was brought in by emergency medical services after being found unconscious on the street. During transport, he was alert and oriented only to the person. He was non-verbal and could not provide further information. Upon arrival at the emergency room, the patient began actively seizing with blood coming from his mouth. The seizure followed a generalized tonic-clonic pattern, lasted two minutes, and spontaneously abated. A 0.5 cm laceration was noted on the left lateral tongue with minimal bleeding.

The patient exhibited a blood pressure of 145/98 mmHg, a mean arterial pressure of 113 mmHg, a heart rate of 87 beats per minute, a respiratory rate of 18 breaths per minute, an oxygen saturation of 100%, and a temperature of 101.1 °F. Physical exam revealed ecchymosis over the right maxilla and a 1 cm laceration anterior to the ear with mild bleeding. Oral exam showed poor dentition and a tongue laceration. Neurological examination showed no focal neurological deficits, no pathological reflexes, no nuchal rigidity, and a negative straight leg test with full range of motion in all four extremities. Relevant initial laboratory investigations are shown in Table 1 and were significant for pronounced hypocalcemia and hypoparathyroidism, an upper limit of normal phosphorus level, and normal vitamin D levels.

**Table 1:** Relevant lab values

Lab marker	Lab result	Reference range
Calcium (mg/dL)	5.4	8.4-10.2
Magnesium (mg/dL)	1.5	1.6-2.6
Phosphate (mg/dL)	4.7	2.3-4.7
Parathyroid hormone (pg/mL)	6.4	8.7-77.1
Vitamin D (ng/mL)	33.5	30-96
Albumin (g/dL)	3.9	3.5-5.0
Sodium (mmol/L)	138	136-145
Potassium (mmol/L)	4.1	3.5-5.1
Chloride (mmol/L)	102	98-107
Creatinine (mg/dL)	1.0	0.7-1.3
Blood urea nitrogen (mg/dL)	11	7-26
Hemoglobin (g/dL)	13.8	13.1-15.5
Platelet count (K/cmm)	135	130-400
White blood cell count (K/cmm)	6.6	4.8-10.8
Serum alcohol (g/dL)	<0.01	<0.01
Acetaminophen level (mcg/ml)	<3.0	7.0-20.0
Acetylsalicylic acid level (mg/dL)	<5.0	5.0-20.0
Troponin (ng/L)	17	<6

Given the seizures, altered mental status, and fever, meningoencephalitis was high on the differentials. A lumbar puncture was attempted in the emergency department; however, samples were insufficient for analysis. Subsequently, the patient was prophylactically started on an empiric treatment for meningoencephalitis due to new-onset seizure and altered consciousness. The patient was started on acyclovir for 14 days, vancomycin for four days, ceftriaxone for seven days, and dexamethasone for four days.

**Figure 1:**
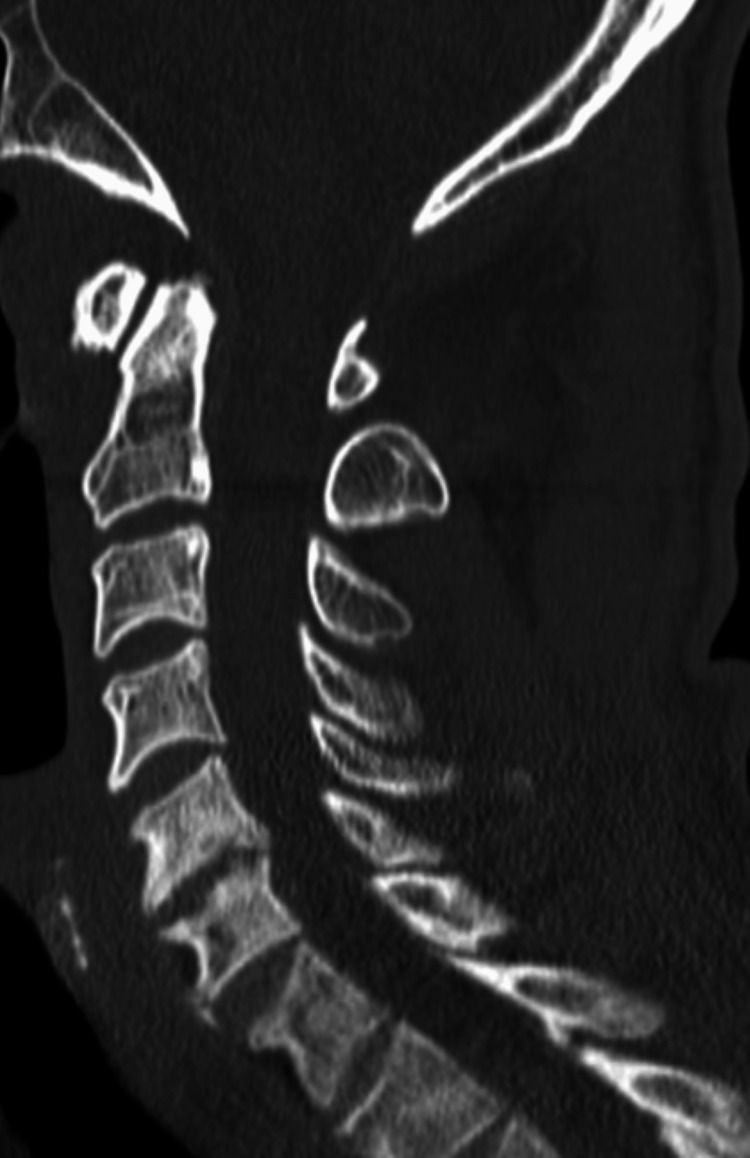
Non-contrast enhanced CT cervical spine showing no acute fractures with only mild degenerative bone changes noted CT: computed tomography

**Figure 2:**
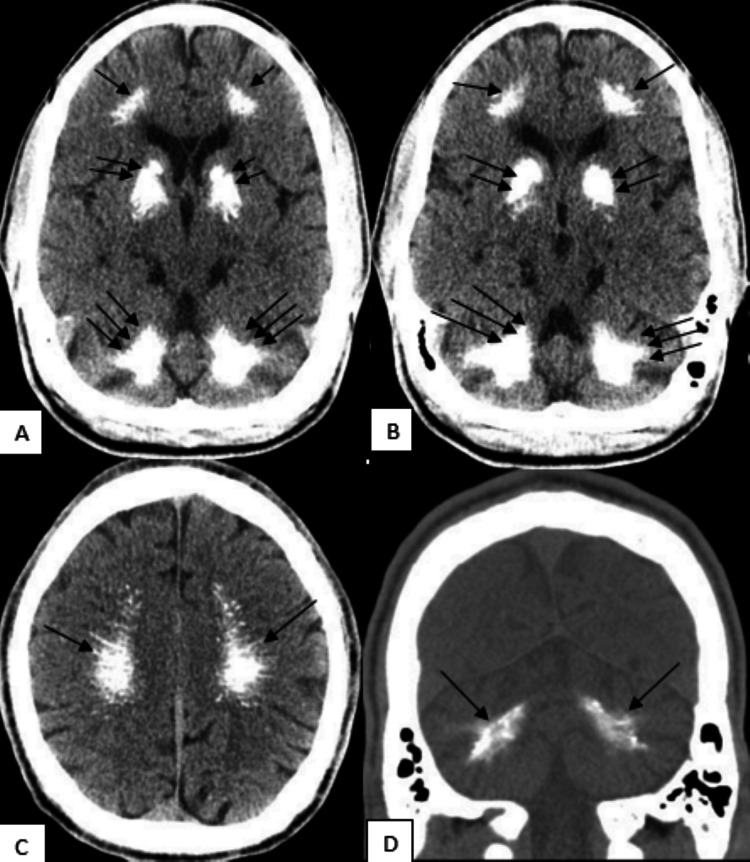
Non-contrast-enhanced CT of the head in axial planes (A-C) and a coronal plane (D) demonstrates large, diffuse, non-punctate calcifications bilaterally A-B: Axial plane CT. Single arrows demonstrate frontal cerebral white matter calcifications. Double arrows demonstrate basal ganglia calcifications, particularly in the globus pallidus region of the basal ganglia. Triple arrows demonstrate cerebellar dentate nuclei calcifications. C: Axial plane CT. Single arrows demonstrate cerebral centrum semiovale calcifications. D: Coronal plane CT. Single arrows demonstrate cerebellar dentate nuclei calcifications. CT: computed tomography

**Figure 3:**
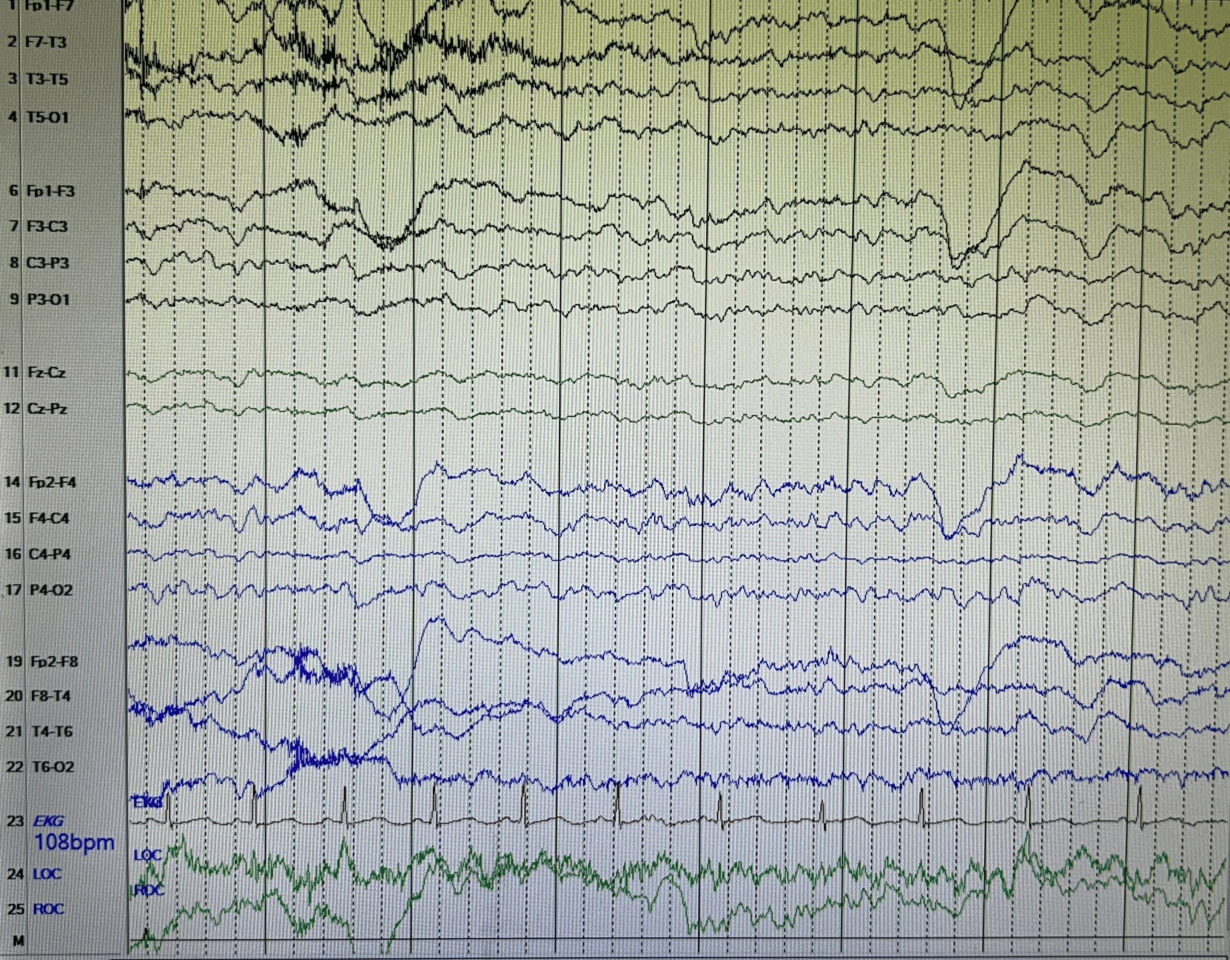
EEG shows diffuse slowing EEG: electroencephalogram

CT cervical spine (Figure 1) showed no evidence of acute fractures, noting some evidence of multilevel degenerative changes. Non-contrast CT head (Figure 2) showed no evidence of acute intracranial hemorrhage; however, it revealed extensive areas of dystrophic calcification in the bilateral basal ganglia, cerebral white matter, and cerebellum. Neurology consult recommended an electroencephalogram (EEG) and to load with levetiracetam. EEG readings (Figure 3) showed diffuse slowing prominent over the bitemporal regions, supporting global cerebral dysfunction. Magnetic resonance imaging (MRI) of the brain was also recommended to further characterize the calcifications and assess for encephalitis; however, the patient declined an MRI.

**Figure 4:**
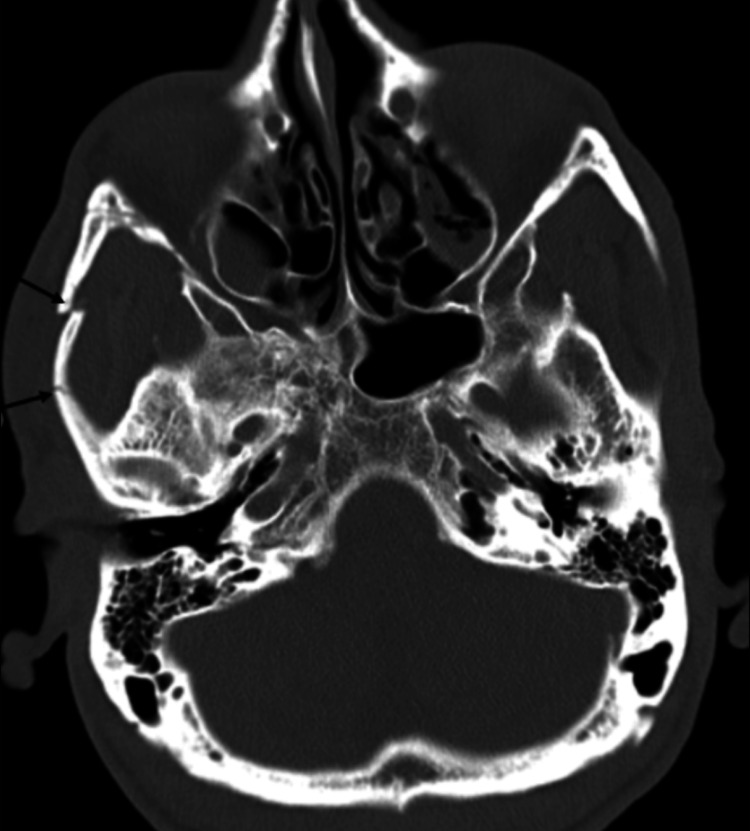
Non-contrast-enhanced CT maxillofacial shows an area with two segmental fractures of the right zygomatic arch (single arrows) CT: computed tomography

A maxillary facial CT (Figure 4) was obtained due to facial trauma and showed acute comminuted, mildly displaced right zygomatic arch fractures. The oral maxillofacial surgical team was consulted, recommending no surgical intervention, administration of augmentin 875/125 mg twice daily for seven days, and outpatient follow-up.

Initially admitted to the ICU for suspected meningoencephalitic encephalopathy, the patient was downgraded to the medical floor after one week, once stable, to continue antibiotic treatment while the causes of intracranial calcifications were further investigated. Endocrinology was consulted for hypocalcemia and recommended starting the patient on vitamin D and calcium supplementation as needed. In addition to calcium supplementation, the patient also received magnesium and potassium supplementation as needed. His symptoms improved clinically, although hypocalcemia and hyperphosphatemia persisted throughout his hospital course. His discharge management plan was initiated, focusing on symptomatic control of hypocalcemia, which included oral calcium carbonate tablets and oral vitamin D capsules. The patient also received levetiracetam tablets for seizure management, and he was recommended to continue quetiapine for psychiatric management. He was discharged to subacute rehabilitation (SAR) due to ongoing functional limitations, including gait instability, intellectual disability, psychiatric disorder, and persistent cognitive impairment. SAR was recommended to provide a safe environment for multidisciplinary rehabilitation, including physical and occupational therapy, and to ensure close monitoring of his hypocalcemia and seizure activity. Additionally, the patient’s limited ability to self-manage medications and lack of reliable social support made SAR the most appropriate setting for continued care and safe transition toward outpatient follow-up. Neurology and endocrinology follow-up appointments were arranged to monitor seizure control and manage long-term metabolic derangements. These appointments were necessary due to the chronic nature of Fahr’s disease, the risk of recurrent seizures, and the need for ongoing titration of calcium and vitamin D therapy. The patient was instructed to follow up with his primary care physician in one to two weeks following discharge.

## Discussion

Fahr’s disease is a neurodegenerative disorder of dystrophic calcium deposition in the brain, which may occur idiopathically or as a result of endocrine or metabolic disorders. This is an exceptionally rare disease affecting less than one in one million people; moreover, this case exhibited a number of unusual features when discerning between an idiopathic Fahr’s disease diagnosis versus Fahr’s disease secondary to an underlying medical condition [6].

Idiopathic Fahr’s is usually autosomal dominant, linked to genes such as *SLC20A2, PDGFB, MYORG*, and others [1,4,8,9]. Due to frequent underdiagnosis and a lack of change in clinical management despite diagnosis, this disease is poorly understood in the literature thus far. The current understanding of the pathophysiological mechanism is that through abnormalities in these genes, there is a disruption in the blood-brain barrier endothelial cells (*IBGC1-4* and *PDFFRB*) or a sodium-dependent phosphorus transporter (*PDGFB* and *SLC20A2*), resulting in abnormal brain calcium deposition [9]. It is important to note that intracranial calcium deposits can be a normal radiologic finding, with studies showing varying degrees of basal ganglia calcifications ranging between 1% and 20% of head CT scans [10,11]. This number would presumably increase on account of other common calcifications correlated with aging in anatomical locations such as the pineal gland, choroid plexus, falx cerebri, and intracranial arteries [12]. In comparison to physiologic or other pathological calcifications, Fahr’s disease calcifications typically are bilaterally symmetric, larger, more diffuse, less punctuate, and found predominantly in portions of the basal ganglia (most commonly in the globus pallidus regions) and to a lesser degree in the cerebellum (most commonly in the dentate nucleus) or cortical/subcortical white matter [9]. The patient’s presentation of particularly large and diffuse calcifications in these locations supports the diagnosis of Fahr’s disease, which is idiopathic by definition, though various features of the patient’s presentation and history may suggest a secondary cause.

The patient’s age falls within the typical symptomatic onset window of Fahr’s disease, usually between 30 and 50 years of age. Family history was unknown due to social restrictions, but the patient’s family reported hallmark symptoms of Fahr’s disease to be present since childhood. These symptoms included gait instability, recurrent seizures, intellectual disabilities, and psychiatric disturbances, which may be logically correlated with the locations in which calcifications were visualized on CT imaging. The childhood-presenting nature of this case instinctively raises the question of whether the radiologic findings are due to a secondary cause related to calcium derangements, such as parathyroid dysfunctions, congenital infections, toxic poisonings, or immune dysfunctions. This patient was persistently hypocalcemic throughout the hospital stay, which, in conjunction with low parathyroid hormone levels and upper limit of normal phosphorus levels, indicated some degree of hypoparathyroidism. Secondary Fahr’s disease has been associated with iatrogenic hypoparathyroidism as a complication of thyroidectomy, but this patient had no such surgical history. This patient showed no signs of immune dysfunction, and a limited social history was unremarkable for any lead poisoning or other toxic causes of intracranial calcifications. Other genetic causes of hypoparathyroidism, such as DiGeorge’s syndrome, familial hypoparathyroidism, and a variety of mitochondrial disorders, are less likely due to a lack of additional clinical abnormalities, but cannot be excluded due to the unknown nature of the patient’s family history. Ultimately, the patient suffered from chronic hypoparathyroidism that was likely longstanding, which led to chronic intracranial calcium deposition with symptoms and radiologic findings consistent with Fahr's disease. 

A CT scan is the gold standard in diagnosing symptomatic Fahr’s disease due to its reliable visibility of calcifications [13]. Calcifications on conventional MRI show varying signal intensities, making it less preferable than CT for diagnosis [13]. A susceptibility‑weighted imaging (SWI) MRI sequence may detect calcifications more clearly and should be integrated with MRI studies if additional imaging is desired [14]. EEG may be useful in evaluating Fahr’s disease presenting with seizures. A thorough clinical assessment of labs is necessary to rule out secondary endocrine, metabolic, toxic, or immunologic causes of Fahr’s disease. 

There is no known cure or disease-modifying agent to treat idiopathic Fahr's disease, though bisphosphonates such as etidronate have been shown to demonstrate potential through inhibition of vascular calcification [15]. Current literature revolves around symptomatic management, with early diagnosis and intervention showing improved patient outcomes by limiting neural tissue damage [16]. Acute hypocalcemia-related CNS hyperexcitability has been well documented to cause seizures, tetanus, and delirium, with 20%-25% of acutely hypocalcemic patients and 30%-70% of idiopathic hypoparathyroid patients exhibiting clinical features of epilepsy [17,18]. Hypoparathyroid-related seizures and movement dysfunctions may be corrected with full symptomatic reversal using alpha-hydroxy vitamin D, calcium supplementation, and corticosteroids [6]. In this case, the childhood onset of this patient’s symptoms and potential long-term hypocalcemia-related tissue damage from dystrophic calcification may limit the extent of symptomatic reversal. Seizures unrelated to metabolic derangements can be managed with typical seizure medications such as levetiracetam.

This patient’s psychiatric symptoms had been managed long-term sufficiently with quetiapine. Atypical antipsychotics and clonazepam yield better clinical outcomes for psychiatric symptoms in these patients [19]. Typical antipsychotics have increased extrapyramidal symptoms, which may exacerbate existing movement and gait symptoms in Fahr’s disease patients [20]. Lithium should be used cautiously due to increased risk for seizures, and carbamazepine, benzodiazepines, and barbiturates may worsen gait disturbances in Fahr’s disease patients [19]. Clinicians should exercise caution when prescribing antidepressants and anxiolytics to patients with Fahr’s syndrome, as they may experience side effects at lower thresholds than unaffected individuals [6].

Intellectual disabilities from this disease are difficult to manage if not addressed quickly, but early treatment has resulted in complete resolution of intellectual disabilities in a three-year-old with secondary Fahr’s disease, showing promise in this regard [21]. Secondary movement disorders due to calcifications in the basal ganglia or cerebellum may be managed with physiotherapy and antipsychotics [22]. Genetic counseling is an important aspect of care in this autosomal dominant inherited disorder. Genetic testing for Fahr’s disease-related genes may enable further diagnostic confirmation; however, it does not influence therapeutic strategy given the lack of curative options. Genetic testing for Fahr’s disease still has some utility in providing valuable insight to at-risk patients when making decisions regarding conception. The possibility of genetic testing in the patient presented was low due to very limited social, financial, and familial support. Pre-symptomatic testing via CT imaging remains ethically contentious, particularly in individuals under 18 years of age, due to the limited clinical utility of a positive or negative result in the absence of a curative treatment [6]. Moreover, the potential psychological harm associated with early diagnosis may outweigh any theoretical benefits [6].

## Conclusions

This case highlights the diagnostic complexity of Fahr’s disease, particularly when clinical and biochemical findings suggest an overlap between idiopathic and secondary forms. While the patient’s extensive, symmetrical brain calcifications and lifelong neuropsychiatric symptoms are characteristic of idiopathic Fahr’s disease, the presence of persistent hypocalcemia and low parathyroid hormone raises concern for a secondary etiology such as long-standing hypoparathyroidism, which was more likely in this case. Differentiating between the two is essential, as secondary causes may offer reversible or manageable treatment options. Diagnosis is best achieved through CT imaging. There is no known cure or disease-modifying agent for Fahr’s disease, though promising new therapies are emerging. Early identification and targeted symptomatic treatment may help improve quality of life and reduce further neurological deterioration. Genetic testing may confirm idiopathic Fahr’s disease, but it is not necessary for diagnosis, nor does it impact treatment strategies. 
